# ^1^H, ^13^C and ^15^N resonance assignments of S114A mutant of UVI31+ from *Chlamydomonas reinhardtii*

**DOI:** 10.1007/s12104-012-9455-1

**Published:** 2012-12-25

**Authors:** Himanshu Singh, Vandana Raghavan, Manish Shukla, Basuthkar J. Rao, Kandala V. R. Chary

**Affiliations:** 1 Department of Chemical Sciences, Tata Institute of Fundamental Research, 1, Homi Bhabha Road, Colaba, Mumbai, 400005 India; 2 Department of Biological Sciences, Tata Institute of Fundamental Research, 1, Homi Bhabha Road, Colaba, Mumbai, 400005 India

**Keywords:** NMR, Resonance assignments, S114A, UVI31+, *Chlamydomonas reinhardtii*

## Abstract

Almost complete sequence specific ^1^H, ^13^C and ^15^N resonance assignments of S114A mutant of UVI31+ from *Chlamydomonas reinhardtii* are reported. The cDNA of S114A mutant of UVI31+ was cloned from a eukaryotic green algae (*C. reinhardtii*) and overexpressed in *E.coli* from where the protein was purified to homogeneity. The point mutation S114A in UVI31+ reduces its DNA endonuclease activity substantially as compared with its wild type. As a prelude to the structural characterization of S114A mutant of UVI31+, we report here complete sequence-specific ^1^H, ^13^C and ^15^N NMR assignments.

## Biological context

Apoptosis in higher multi-cellular organisms plays an important role in homeostasis, development and defence; however its significance in unicellular organisms remains unclear. *Chlamydomonas reinhardtii*, a unicellular green alga, has been shown to undergo apoptosis in response to UV-C irradiation (Kim et al. 1997; Moharikar et al. 2006). In order to understand the process of UV mediated apoptosis in *C. reinhardtii*, we undertook in silico global genome analysis and found a protein, which was identified as UVI31+, one amongst several UV inducible transcripts/proteins and a novel β-lactamase (Moharikar et al. 2006, Rout et al. 2010). UVI31+ protein is also endowed with endonuclease function, in vitro. Cell biological regulation reveals interesting localization changes of UVI31+ protein in *Chlamydomonas reinhardtii* chloroplast compartment (Shukla et al. 2012).

*Chlamydomonas reinhardtii* is about 10 μm in diameter that swims with two flagella. They have a cell wall made up of hydroxyproline-rich glycoproteins, a large cup-shaped chloroplast, a large pyrenoid, and an “eyespot” that senses light (Moharikar et al. 2006). It undergoes apoptosis in response to UV-C irradiation (Moharikar et al. 2007). It shows classical hall-marks of animal cell apoptosis and hence can be used as a model system for studying its molecular mechanism in a plant-like environment. Certain candidate molecules were recently identified as to be either UV-regulated or involved in apoptosis. They include apoptosis protease activating factor (APAF), a caspase-3 like protein and a defender against apoptotic death (*dad1*). All of them exhibited a distinct activation pattern correlating with onset of death following UV irradiation (Moharikar et al. 2006, 2007). One of the putative candidate molecules that is yet to be characterized is the aforementioned UV inducible gene, *uvi31*+, the focus of the present study.

*Uvi31*+ was originally isolated from *Schizosaccharomyces pombe* (Lee et al. 1994), whose expression was unaltered by other DNA damaging or cytotoxic agents. Interestingly, *uvi31*+ showed no significant sequence homology to the known DNA repair genes. It was observed that *uvi31*+ transcript increases during normal cell cycle in G1 phase before septation and also during diauxic shift (Kim et al. 1997). A null mutant of *uvi31*+ from *S. pombe* showed sensitivity to UV-light, defects in septation and cytokinesis during the resumption of cell division following UV damage-induced cell cycle arrest (Kim et al. 2002).

The *uvi31*+ protein has structural homology with the BolA protein of *Escherichia coli,* which was identified by its ability to induce round cell morphology when over-expressed in cells and also following a general stress response (Aldea et al. 1988; Santos et al. 1999; Huynen et al. 2005). Recently, it has been found that *uvi31*+ exhibited endonuclease activity (Shukla et al. 2012). However, *uvi31*+ has no structural homology with the known endonucleases. The mutation at S114A site at *uvi31*+ reduced its endonuclease activity as derived from biochemical analysis (yet to be published). And it has also been speculated that the Ser residue (S114) may be responsible for this activity. With this in the backdrop, we have mutated Ser114 to Ala114 and set out to structurally characterize the mutant of *uvi31*+ (named as S114A*uvi31*+) by NMR spectroscopy, to find out whether S114 is involved in endonuclese activity. Towards this goal, we report almost complete sequence specific ^1^H, ^13^C and ^15^N NMR assignments of S114A mutant of UVI31+.

## Methods and results

Cloning, over-expression and purification of UVI31+ were carried out as described earlier (Rout et al. 2010). ^13^C_6_-glucose or/and ^15^NH_4_Cl (Cambridge Isotopes Inc.) were used as the sole source of carbon or/and nitrogen. The purification of the protein was achieved by Ni^++^-NTA (Ni^++^- nitrilotriacetate) agarose (Qiagen, Hilgen, Germany). His6-tagged S114A mutant of UVI31+ was eluted with 250 mM imidazole in 50 mM sodium phosphate (pH 7.6), 100 mM NaCl. The eluted fractions were dialyzed overnight against 50 mM sodium phosphate (pH 6.4), 100 mM NaCl.

For NMR experiments, protein samples were prepared in a mixed solvent of 90 % H_2_O and 10 % ^2^H_2_O containing 50 mM sodium phosphate (pH 6.4) and 100 mM NaCl. The protein concentrations were 0.8 mM. NMR experiments were recorded on a Bruker Avance 800 MHz NMR spectrometer equipped with a 5 mm triple-resonance cryogenic probe. Experiments recorded at 298 K with uniformly ^15^N-labled-S114A mutant of UVI31+ included sensitivity-enhanced 2D [^15^N–^1^H]–HSQC using water-flipback for minimizing water saturation (Bax et al. 1991). The 3D experiments recorded with doubly (^13^C and ^15^N) labeled samples were, HNCO, HN (CA)CO, CBCANH, and CBCA(CO)NH (Bax et al. 1991; Bax and Grzesiek 1993; Wuthrich et al. 1986), essentially for the assignment of backbone resonances. For the assignment of theThe side chain ^1^H and ^13^C resonances, we recordeds were assigned using 3D C(CO)NH and 3D H(CCO)NH (Montelione et al. 1992) and ^13^C- and ^15^N-edited NOESY spectra (Muhandiram et al. 1993). NMR data processing was carried out using Bruker Topspin 3.1 software and analyzed with TATAPRO (Atreya et al. 2000, 2002) and CARA (Keller 2002). ^1^H chemical shifts were referenced with respect to the external standard 2,2-dimethyl-2-silapentene-5-sulfonates (DSS), while ^15^N and ^13^C chemical shifts were calibrated indirectly (Edison et al. 1994; Chary and Govil 2008).

## Extent of assignment and data deposition

**Fig. 1:**
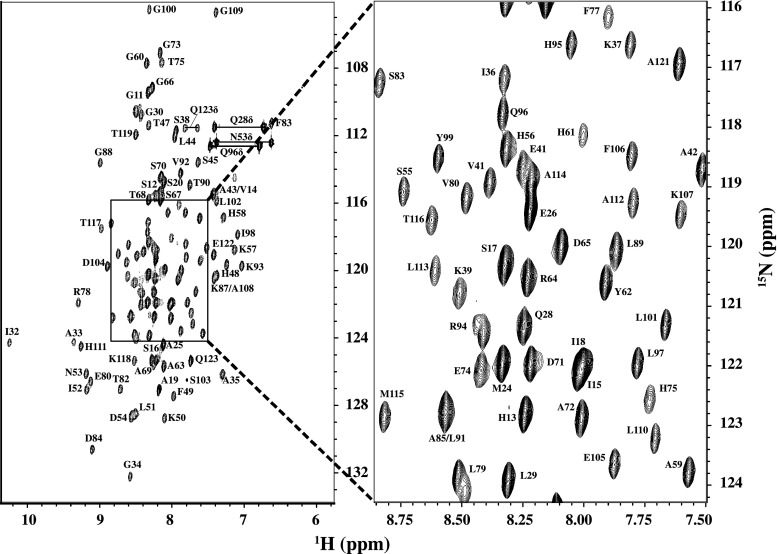
2D [^15^N–^1^H]–HSQC of S114A mutant of UVI31+ at pH 6.4 and 298 K with assignments. The spectrum was recorded with 256 and 1,024 complex points along t_1_ and t_2_ dimensions, respectively. The assignments are indicated by the *one-letter* amino acid code followed by the corresponding sequence number along the protein primary sequence. The *peaks* connected with *horizontal lines* are correlations from sidechain NH_2_ spin pairs belonging to Asn and Gln residues

**Fig. 2:**
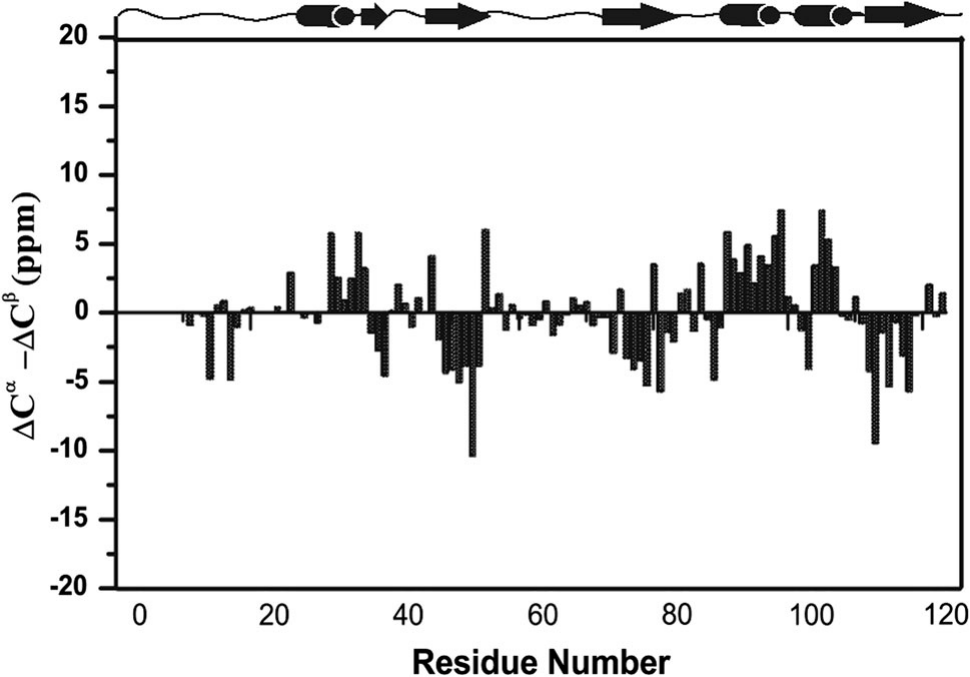
The secondary structural characterization as derived from the difference between the ∆C^α^ and ∆C^β^ values (∆C^α^−∆C^β^) of S114A mutant of UVI31+. The value ∆C^α^−∆C^β^ for a particular residue i represents the average over the three consecutive residues, i−1, i and i + 1. A stretch, having *negative* values of ∆C^α^−∆C^β^, indicates the presence of extended β-strand whereas *positive* values indicate a regular α-helical region

Sequence specific resonance assignments of S114A mutant of UVI31+, which is free of Cys and Trp, could be carried out for nearly all ^1^H, ^13^C and ^15^N spins using a suite of 3D NMR experiments mentioned in Methods. The ^1^H^N^ and ^15^N assignments thus derived are shown in the 2D [^15^N–^1^H]–HSQC (Fig. 1). A total of 117(^1^H_i_, ^13^C_i−1_,^15^N_i_) correlations are expected from non-proline residues in the 3D-HNCO spectrum of S114A mutant of UVI31+ that has a His-tag of 6 residues. We could however observe 107 distinct correlations and assign almost all of them (97 %) under the experimental conditions described herein. In addition, the side chain ^1^H and ^13^C were assigned for most of the residues (88 % of aliphatic ^1^H resonances and 92 % aliphatic ^13^C resonances) with an exception of aromatic resonances. The peaks connected by horizontal lines in Fig. 1 belongs to the pairs of side chain NH_2_ correlations belonging to Asn and Gln residues. The chemical shift data thus obtained has been deposited in the BioMagResBank (http://www.bmrbwisc.edu) under accession number 18567. Sharp [^15^N–^1^H] peaks of almost uniform intensity and large dispersion (of 4.4 ppm) of resonances along the ^1^H^N^ dimension (Fig. 1) revealed that the protein is in a well folded state. The secondary structure elements (Fig. 2) of the protein have been characterized using the well known empirical relations of ^13^C chemical shifts (∆C_α_−∆C_β_) (Spera and Bax 1991; Wishart et al. 1995; Barnwal et al. 2008). The CSI plot (Fig. 2) reveals the presence of both α-helical and β-strand segments separated by short stretches of unstructured elements. Comparison of [^15^N–^1^H]–HSQC spectra of (S114A)-UVI31+ with that of wild type-uvi31+, both recorded under similar experimental conditions reveal subtle spectral changes between them, though most of the ^15^N–^1^H peaks are unperturbed, suggesting that the overall structural topology of the protein may remain same with substantial conformational changes in the structure particularly near and around the S114A point mutation. Complete 3D structural characterization of the mutant UVI31+ will throw more light on the intricate structural changes caused by the point mutation. This work is in progress.
